# Multiomics Analysis of Nucleotide Metabolism Highlights the Important Role of Adenylate Kinase 4 in Pancreatic Cancer

**DOI:** 10.1155/humu/7729933

**Published:** 2026-05-03

**Authors:** Jun Li, Yiqun Yao, Wei Zhang, Dianlong Zhang

**Affiliations:** ^1^ Department of Breast and Thyroid Surgery, The Affiliated Zhongshan Hospital of Dalian University, Dalian, Liaoning, China; ^2^ Department of Neurosurgery, The First Affiliated Hospital of Dalian Medical University, Dalian, Liaoning, China, dlmedu.edu.cn; ^3^ State Key Laboratory of Structural Analysis, Optimization and CAE Software for Industrial Equipment, School of Mechanics and Aerospace Engineering, Dalian University of Technology, Dalian, Liaoning, China, dlut.edu.cn

**Keywords:** adenylate kinase 4, metabolomics analysis, nucleotide metabolism, pancreatic cancer, RNA-seq analysis, single-cell sequencing analysis, spatial transcriptomics analysis

## Abstract

Nucleotide metabolism significantly influences tumor cell proliferation, yet its specific profile in pancreatic cancer remains inadequately understood. This study was aimed at characterizing the nucleotide metabolic profile in pancreatic cancer and assessing the contribution of the key gene adenylate kinase (AK) 4. Multiomics data, including transcriptomic, single‐cell sequencing, spatial transcriptomic, and metabolomics datasets, were obtained from publicly accessible platforms. The impact of AK4, a key gene of nucleotide metabolism, on the proliferation and migration of pancreatic cancer cells was investigated using various molecular biological techniques. Nucleotide pathway–related metabolites exhibited marked differences in abundance between pancreatic cancer tissues and normal pancreatic tissues. Single‐cell sequencing analysis identified MKI67^+^ and myeloid cells as subsets with overactive nucleotide metabolism. Immune cells from tumor tissues had a higher score of nucleotide metabolism than those from the normal pancreas. Spatial transcriptomics revealed spatial features of nucleotide metabolism in pancreatic cancer. Pancreatic cancer patients displayed distinct clinical heterogeneity in nucleotide metabolism, with elevated nucleotide signaling correlating with poorer patient prognosis. Furthermore, tumor subtypes showed variations in immune microenvironment features and immune checkpoint expression, which may explain their differential prognoses. A nucleotide metabolic–derived prognostic panel had the potential to predict the clinical outcomes of patients with pancreatic cancer. The AK4 gene played a central role in nucleotide metabolism, and its overexpression in clinical pancreatic cancer samples was frequently linked to adverse patient outcomes. Cell‐based experiments revealed that AK4 knockdown suppressed pancreatic cancer cell proliferation and migration. Abnormal nucleotide metabolism pathways are implicated in pancreatic cancer onset and progression.

## 1. Introduction

Pancreatic cancer, often referred to as the “king of cancer,” represents a malignancy of the digestive system with the worst prognosis and the poorest postsurgical quality of life [1, 2]. Notably, the disease typically presents with an insidious onset and is challenging to detect in its early stages [3]. A significant proportion of patients are diagnosed in the advanced stages [4], frequently precluding surgical intervention and imposing substantial social and familial burdens [5]. In the global effort to combat pancreatic cancer, researchers have explored the disease from multiple angles, including the creation of a novel staging system [6], the development of early diagnostic strategies [7, 8], the incorporation of neoadjuvant chemotherapy approaches [9], and the advancement of radiomics‐based predictive models [10, 11]. Despite these extensive endeavors, pancreatic cancer remains a formidable malignancy, with its notorious status as the “king of cancer” firmly entrenched in the public consciousness. The uncontrolled proliferation of tumor cells and other malignant behaviors demands significant energy and base support, with abnormal activation of nucleotide metabolism frequently serving as a key driver for the abnormal proliferation of these cells.

Nucleotide metabolic pathways are frequently reprogrammed to support the rapid proliferation and chemotherapy resistance of pancreatic cancer cells [12]. These cells exhibit a strong reliance on the de novo synthesis pathway to meet the demands of their accelerated growth. Adenylate kinase (AK) serves as a critical regulatory enzyme in high‐energy phosphorylation transfer reactions within the cells of various organisms, playing an essential role in adenine nucleotide metabolism and homeostasis maintenance [13]. Nine isoenzymes of the AK family (AK1–AK9) have been identified in vertebrates to date. This monomeric phosphotransferase catalyzes reversible phosphoryl transfer reactions and modulates intracellular ATP levels [14]. AK is integrally involved in controlling tumor cell metabolism and metabolic signaling, thereby linking metabolic reprogramming to aggressive behaviors like migration and invasion. AK4, in particular, is involved in hypoxia tolerance, resistance to anticancer drugs, and the regulation of mitochondrial activity, positioning it as a potential target for cancer therapies [15, 16]. Recent studies have highlighted the AK4 gene′s role in promoting tumor cell proliferation and metastasis, particularly in bladder [17], lung [18, 19], and ovarian cancers [20]. However, its expression profiles and tumor‐promoting effects in pancreatic cancer remain unclear.

This study investigates the altered nucleotide metabolic activity in pancreatic cancer, integrating multiomics data from metabolomics, single‐cell omics, and transcriptomics. The accumulation of nucleotide‐related metabolites in the tumor microenvironment (TME) may drive the malignant behaviors of cancer cells. Furthermore, distinct nucleotide metabolism profiles exist across pancreatic cancer patients, offering potential for their integration into clinical classification systems, thereby advancing precision oncology. Machine learning has identified AK4 as a key gene involved in nucleotide metabolism. This study demonstrates that elevated AK4 expression in pancreatic cancer correlates with a poor patient prognosis. Additionally, knockdown of AK4 expression attenuates the proliferative and migratory capacities of pancreatic cancer cells.

## 2. Materials and Methods

### 2.1. Metabolomics Data Analysis

Liu et al. [21] performed metabolomics sequencing on 35 pancreatic cancer and 31 normal pancreatic samples, with the results made publicly available. The metabolomics data used in this study were sourced from their report. The data was organized, focusing on the expression abundances of metabolites associated with purine and pyrimidine metabolism. A subsequent comparison was made between the nucleotide metabolism abundances in cancerous and normal tissues.

### 2.2. Single‐Cell Sequencing and Spatial Transcriptomics Data Analysis

The single‐cell sequencing data were obtained from the GSE205049 [22] cohort, which includes sequencing information for nine pancreatic cancer samples and their corresponding adjacent tissues. The Seurat R package′s CreateSeuratObject function was employed to process the single‐cell data, with the parameters min.cells = 3 and min.features = 200. For further data cleaning, additional parameters were applied: nCount_RNA ≥ 1000, nFeature_RNA ≥ 500, nFeature_RNA ≤ 8000, and percent.mt ≤ 20. Standardization and normalization were performed using the NormalizeData and ScaleData functions, while batch effects were removed with the RunHarmony function [23]. Dimensionality reduction and clustering were executed with a resolution parameter of 1.5. The DimPlot function was then used to generate the UMAP visualization. Cell subsets for each cluster were identified based on prior single‐cell annotation methods [24], including acinar, ductal, endocrine, and immune cells, among others. Nucleotide metabolism–related genes were obtained from the MSigDb database (https://www.gsea-msigdb.org/gsea/msigdb/cards/REACTOME_METABOLISM_OF_NUCLEOTIDES).

To quantify the nucleotide metabolism profiles of each cell, methods outlined in previous studies [25, 26] were adopted, incorporating Add, AUCell, UCell, singscore, and ssgsea. To mitigate the potential influence of algorithmic variation, quantitative indicators from these methods were normalized and aggregated, yielding a total score (Scoring), which provided a more stable reflection of cellular metabolic activity. Finally, the Wilcoxon test was applied to assess the differences in nucleotide metabolism activity between cells from distinct sources. Pseudotime analysis [27] was employed to explore the dynamic variation of nucleotide metabolism during the malignant transformation of ductal epithelial cells. Cell–chat analysis [28] was utilized to explore the crosstalk among different cell types. In addition, the GEO platform provided three patients with spatial transcriptomics data. The Seurat package was applied to carry out spatial transcriptomics analysis. Similarly, we also employed multiple gene set enrichment analysis algorithms (i.e., Add, AUCell, singscore, ssgsea, UCell, and total scores) to quantify the nucleotide metabolism features across spatial regions.

### 2.3. Transcriptome Sequencing Data Analysis

The transcriptome sequencing data of 930 samples with pancreatic cancer were obtained from the GSE57495 [29], GSE28735 [30], TCGA‐PAAD, GSE62452 [31], MTAB‐6134 [32], ICGC‐CA, and ICGC‐AU datasets. Unsupervised clustering of TCGA‐PAAD cohort samples, based on the nucleotide metabolism expression profile, identified two metabolic subtypes: C1 and C2. Survival differences between these subtypes were assessed using Kaplan–Meier (KM) curves. The GSVA algorithm was employed to evaluate nucleotide metabolism activity in each pancreatic cancer sample, with the Wilcoxon test applied to assess metabolic differences between the C1 and C2 subtypes. Clinical data from TCGA, including stage, grade, and age, were integrated with metabolic subtyping to explore the clinical characteristics associated with distinct metabolic states. Furthermore, HALLMARK‐related signaling pathways, essential for tumorigenesis and progression, were retrieved from the MSigDb platform [33]. The GSVA algorithm was used to compute HALLMARK scores for each patient, with score differences between C1 and C2 subtypes subsequently compared.

The immune microenvironment remains a central focus in tumor research [34]. To this end, immune characteristics were assessed under the two metabolic states, C1 and C2. Initially, the estimate algorithm [35] was employed for a broad evaluation, quantifying stromal score, immune score, and tumor purity in each pancreatic cancer patient. These metrics provide a general view of the immune landscape. Subsequently, a comprehensive immune prediction algorithm, including TIMER and CIBERSORT [36, 37], was applied to accurately assess the infiltration of various immune cell types. Finally, recognizing the significance of tumor heterogeneity and chemosensitivity, the oncopredict package was utilized to predict drug susceptibility, aiming to identify potential therapeutic agents tailored to individuals with distinct metabolic profiles [38].

Six hundred and thirty‐five patients from the TCGA database, the GEO database, and the ArrayExpress database were identified, and 295 patients from the ICGC database were also obtained for further model construction. Fifty percent of the samples of 635 patients were identified as the training cohort, and the remaining samples were considered the test1 cohort. Additionally, all 635 patients were considered as the test2 cohort. Also, 295 patients were considered the test3 cohort. LASSO‐Cox regression analysis was utilized to filter essential genes and develop a prognostic panel. Survival curves and ROC curves were employed to determine the predictive accuracy.

### 2.4. Prediction of Key Genes Associated With Nucleotide Metabolism Pathways

The random forest algorithm was applied to evaluate the importance of genes involved in the nucleotide metabolism pathway in pancreatic cancer. Genes with higher predicted coefficients were deemed more influential. The Biomarker Exploration for Solid Tumors platform [39] was a publicly available online resource that offers transcriptomics data and clinical information for nearly all human cancers. This platform enables the investigation of potential associations between genes and disease phenotypes. It was used in this study to assess the expression profiles, clinical relevance, and prognostic implications of the key gene AK4 in nucleotide metabolism.

### 2.5. Cell Culture

Pancreatic cancer cell lines employed in this study included Panc‐1, BxPc‐3, and SW1990 cells. BxPc‐3 cells were maintained in 1640 medium supplemented with 10% FBS, while Panc‐1 and SW1990 cells were cultured in DMEM medium with 10% FBS. Prior to passaging, all cells were trypsinized. Cultures were maintained in a 37°C incubator with 5% CO_2_.

### 2.6. RNA Extraction, RNA Reverse Transcription, and PCR Assays

Following cell centrifugation, the pellet was collected and resuspended in 1 mL of TRIzol, ensuring uniform mixing. After standing, chloroform was added, and the mixture was centrifuged according to the manufacturer′s protocol. The resulting supernatant was transferred, and isopropanol was introduced to facilitate RNA precipitation. A subsequent centrifugation step yielded the RNA precipitate, which was then washed with 80% ethanol and centrifuged again. After drying, the RNA pellet was dissolved in enzyme‐free water for concentration measurement. Reverse transcription was carried out as per the RNA reverse transcription kit guidelines. Quantitative PCR analysis was performed using the SYBR method. The primer sequences are shown in Supporting Information 4: Table S1.

### 2.7. Cell Transfection

AK4‐knockdown siRNA reagents and the siRNA Transmate Plus transfection reagent were purchased from GenePharma. The gene silencing sequences are shown in Supporting Information 4: Table S2. Following the manufacturer′s protocol, SW1990 and PANC1 cells were transfected for AK4 gene knockdown, and knockdown efficiency was assessed via PCR analysis.

### 2.8. CCK8 Assay

SW1990 and PANC1 cells in the logarithmic growth phase were utilized for the CCK8 assay. Prior to the experiment, SW1990 and PANC1 cells were seeded in six‐well plates, followed by AK4 knockdown as described earlier. After successful transfection, cells were trypsinized, and 5000 cells were plated into 96‐well plates. Two groups, siNC and siRNA‐AK4, were established. CCK8 reagent was added at 48 h, and absorbance was measured.

### 2.9. Wound Healing Assay

Following successful transfection of the AK4 gene, a 200 *μ*L pipette tip was used to create a “grid” pattern in the six‐well plate for scratching. Cell migration was assessed by capturing images at 0, 24, and 48 h postscratching using an optical microscope, allowing for comparison of the effects of AK4 knockdown on cell migration.

### 2.10. Transwell Migration Assay

Prepare serum‐free culture medium to hydrate the basement membrane before the formal experiment to ensure an appropriate cell migration environment. An appropriate amount of cells was placed in the upper chamber of the transwell chamber, while medium containing 10% serum was added to the lower chamber to stimulate their migration. After 24 h, crystal violet staining was used, and micrographs were taken.

### 2.11. Immunohistochemical Assay

Human pancreatic cancer tissue microarrays and corresponding ethical certificates were obtained from Shanghai Weiao Biological Company. AK4 antibodies for immunohistochemistry were purchased from Affinity Biosciences (Cat# DF6781). The procedure was as follows: The tissue microarray was immersed in xylene and varying ethanol concentrations, then transferred to a boiling antigen retrieval solution. After thorough PBS washing, the chip was treated with a 3% hydrogen peroxide solution to block endogenous peroxidase activity. Following serum blocking with goat serum, the chip was washed with PBS, incubated with AK4 antibody overnight at 4°C, and processed the next day. Immunohistochemical staining was performed, after which the chip was sealed and photographed.

## 3. Results

### 3.1. Metabolism Characteristics of Nucleotide‐Associated Metabolites in Pancreatic Cancer

**Figure 1:**
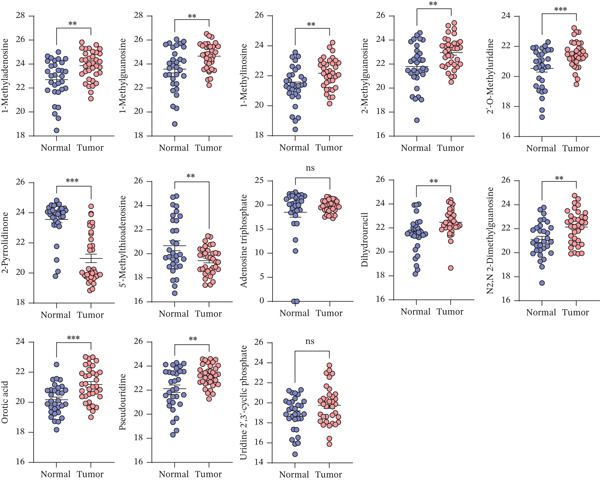
Abundance of nucleotide pathway–related metabolites in pancreatic cancer tissues compared to normal pancreatic tissues. ns, not significant;  ^∗∗^ *p* < 0.01 and  ^∗∗∗^ *p* < 0.001.

The nucleotide metabolic profile of pancreatic cancer tissue deviated significantly from that of normal pancreatic tissue, primarily characterized by an abnormally increased nucleotide metabolite. Nucleotide metabolism plays a crucial role in facilitating malignant behaviors such as cancer cell proliferation and migration. Metabolites, including 1‐methyladenosine, 1‐methylguanosine, 1‐methylinosine, 2‐methylguanosine, 2‐pyrrolidinone, dihydrouracil, N2, N2‐dimethylguanosine, orotic acid, and pseudouridine, exhibited markedly elevated levels in pancreatic cancer tissue. Conversely, metabolites such as 2‐pyrrolidinone and 5 ^′^‐methylthioadenosine showed a significant decrease in abundance (Figure 1).

### 3.2. Characterizing Nucleotide Metabolism in Pancreatic Cancer Using Single‐Cell and Spatial Transcriptomics

**Figure 2:**
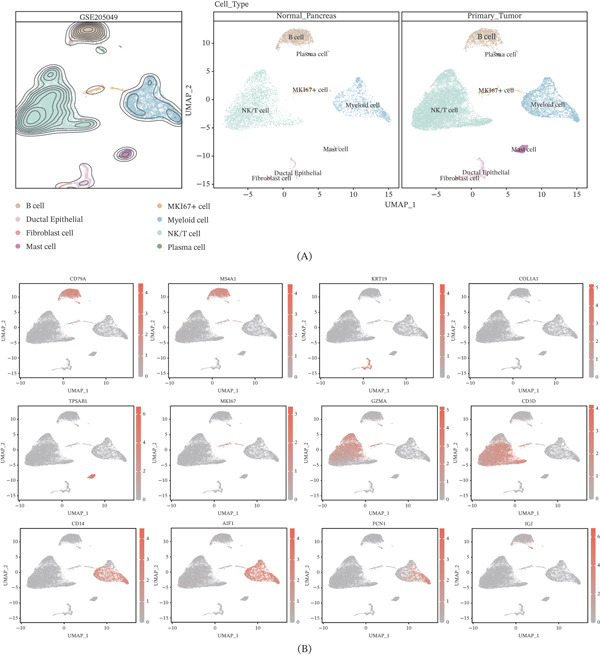
Single‐cell atlas of pancreatic cancer. (A) UMAP dimensionality reduction plot of pancreatic cancer cell subpopulations. (B) Expression distribution of marker genes in pancreatic cancer cell subpopulations.

Following quality control, a total of 25,788 single cells were selected for subsequent omics analysis. After performing UMAP dimensionality reduction, 25 distinct cell subsets were identified (Supporting Information 1: Figure S1). These subsets were then annotated using marker genes, with the following classifications: Subsets 4, 6, and 23 corresponded to B cells; Subsets 17 and 20 were classified as pancreatic ductal epithelial cells; and Subset 15 was identified as mast cells; Subsets 22 and 24 as plasma cells; Subsets 8, 11, 12, 13, 14, and 19 as myeloid cells; Subset 21 as fibroblasts; Subset 18 as MKI67^+^ cells; and Subsets 0, 1, 2, 3, 5, 7, 9, 10, and 16 as NK/T cells. As illustrated in Figure 2A, a total of eight subsets were identified, with clear groupings and precise identification. To better visualize the marker gene expression for each cell group, the spatial expression of each marker was mapped, as shown in Figure 2B. CD79A and MS4A1 were the primary markers for B cells; KRT19 for ductal epithelial cells; COL1A1 for fibroblasts; TPSAB1 for mast cells; GZMA and CD3D for NK/T cells; CD14, AIF1, and FCN1 for myeloid cells; and IGJ for plasma cells.

**Figure 3:**
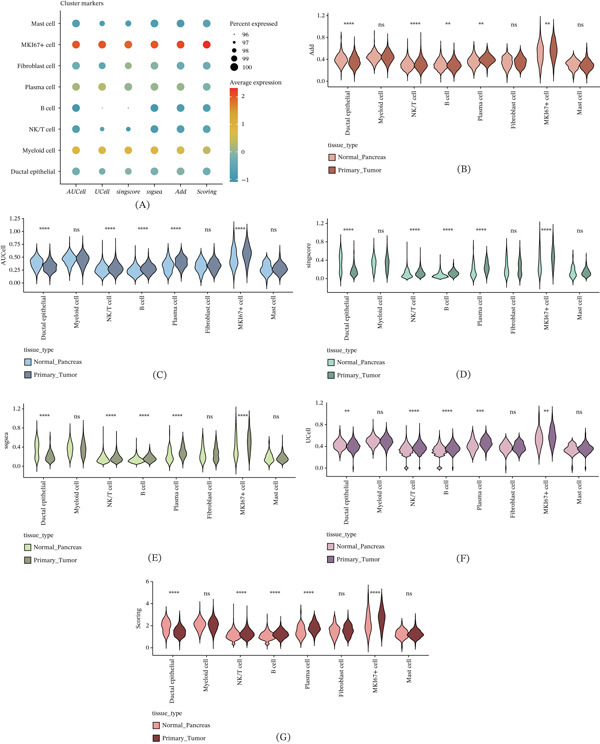
Analysis of nucleotide metabolism characteristics in pancreatic cancer at single‐cell resolution. ns, not significant;  ^∗∗^ *p* < 0.01,  ^∗∗∗^ *p* < 0.001, and  ^∗∗∗∗^ *p* < 0.0001. (A) Nucleotide metabolism characteristics of various cell subpopulations in pancreatic cancer. Prediction of nucleotide metabolism characteristics in different cell types of cancerous and normal pancreatic tissues using the (B) Add algorithm, (C) AUCell algorithm, (D) singscore algorithm, (E) ssgsea algorithm, (F) UCell algorithm, and (G) Scoring algorithm.

As illustrated in Figure 3A, nucleotide metabolism was most pronounced in MKI67^+^ and myeloid cells. The metabolic differences across various cell subsets in the normal pancreas and pancreatic cancer were then assessed. The Add (Figure 3B), AUCell (Figure 3C), singscore (Figure 3D), ssgsea (Figure 3E), UCell (Figure 3F), and Scoring (Figure 3G) algorithms revealed that multiple cell types within the pancreatic cancer microenvironment exhibited heightened nucleotide metabolism activity, including NK/T cells, B cells, and MKI67^+^ cells. In contrast, nucleotide metabolism signals in ductal epithelial cells were notably weaker. Although pancreatic ductal epithelial cells serve as the primary site of pancreatic cancer lesions, their nucleotide metabolism should theoretically be more active due to the high demand for purine and pyrimidine pools in cancer cell proliferation. However, this study observed the opposite. This discrepancy may be attributed to the limited number of ductal epithelial cells within the single‐cell cohort. The UMAP dimensionality reduction plot clearly showed a relatively low proportion of ductal cells, which may explain the observed result. Further extensive sequencing studies are necessary to investigate this further. Finally, to better visualize the nucleotide metabolic profiles under each algorithm, the quantitative results were mapped onto the UMAP visualization (Supporting Information 2: Figure S2).

**Figure 5:**
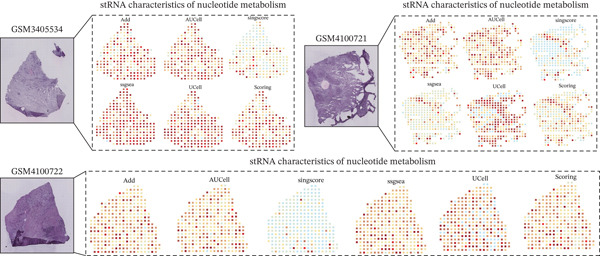
Spatial transcriptomics features of nucleotide metabolism in pancreatic cancer.

The nucleotide metabolism–related genes undergo corresponding changes with the occurrence of ductal epithelial cell carcinogenesis (Figure 4B). We observed that genes such as TYMP, AK5, and ADK were highly active in the early stages of the lesion (Figure 4A), suggesting their potential involvement in the early pathogenesis of pancreatic cancer, such as ADM and PanIN. In contrast, genes like UPP2 and AK4 showed relatively higher activity during the intermediate stages of the lesion, implying their possible role in maintaining tumor phenotypes and promoting tumor progression (Figure 4A). In the late stages of the lesion, AK2 and AK7 exhibited relatively increased expression, indicating their potential association with tumor invasion and even advanced cachexia (Figure 4A). Figure 4C illustrates the cell–cell communication network between ductal cells and other cells under different nucleotide metabolic states. Figure 4D displays the ligand–receptor relationships involved in these cellular communications. Spatial transcriptomics features of nucleotide metabolism are plotted in Figure 5.

Figure 4Cell trajectory and cell–cell communication analysis. (A) The heatmap shows the dynamic variation of nucleotide metabolism. (B) Cell trajectory plots of nucleotide metabolism scores. (C) Cell–chat network. (D) Ligand–receptor interaction network.

**Figure:**
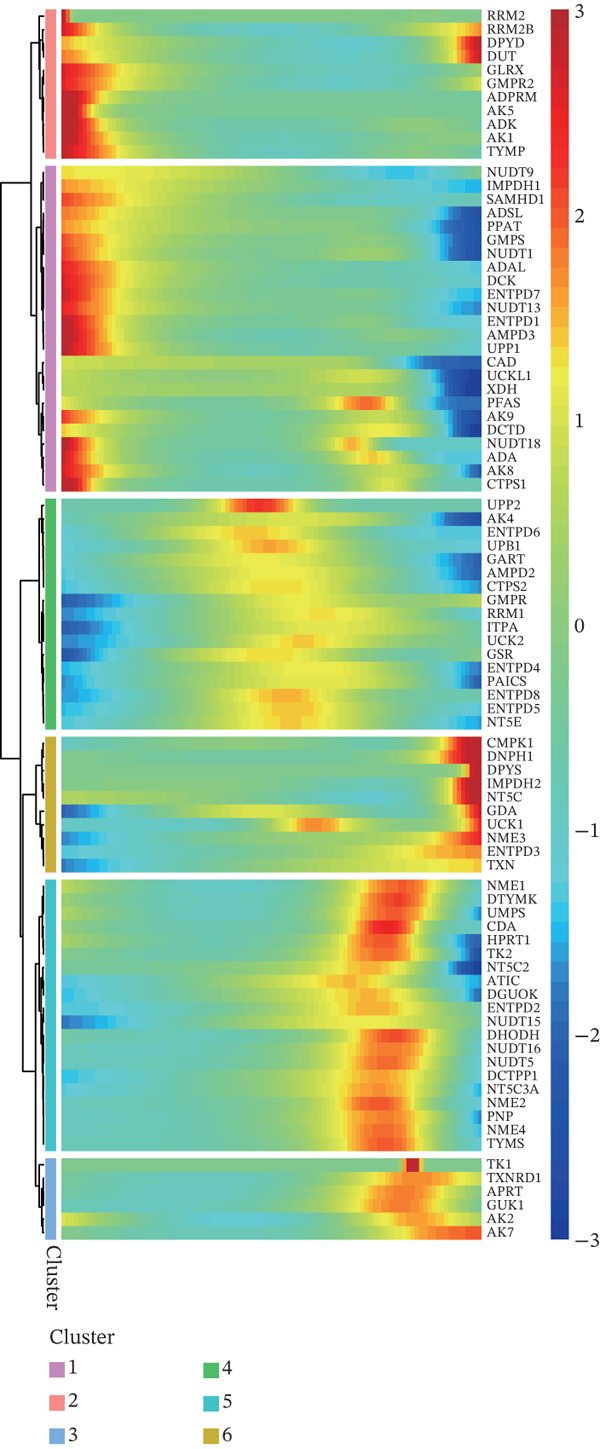
(A)

**Figure:**
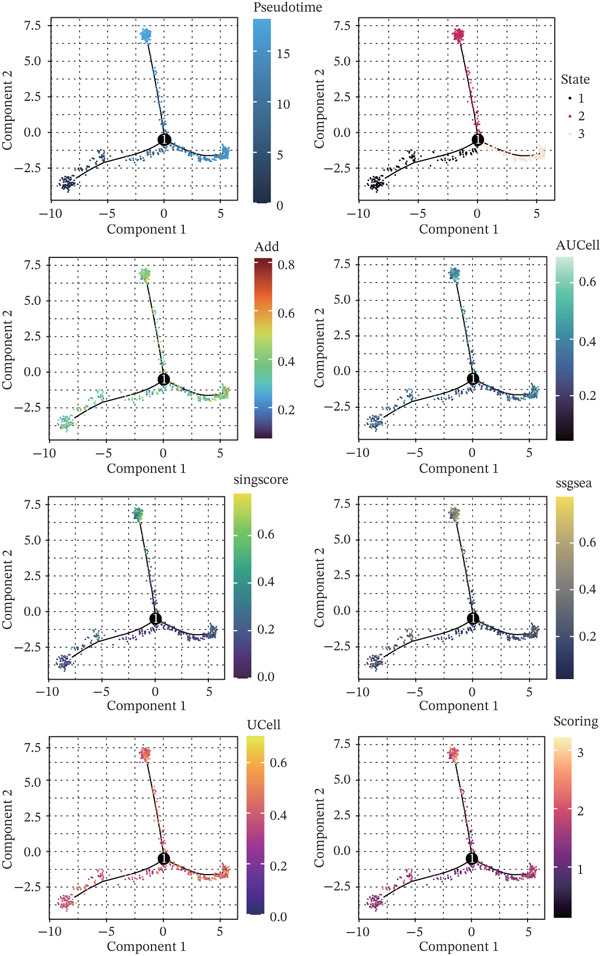
(B)

**Figure:**
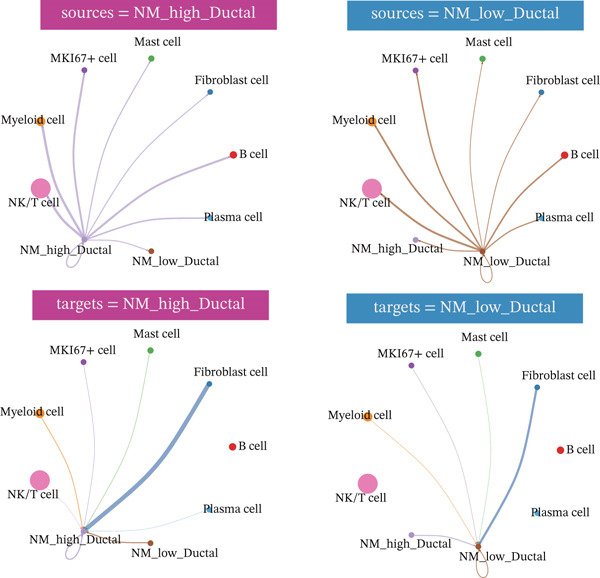
(C)

**Figure:**
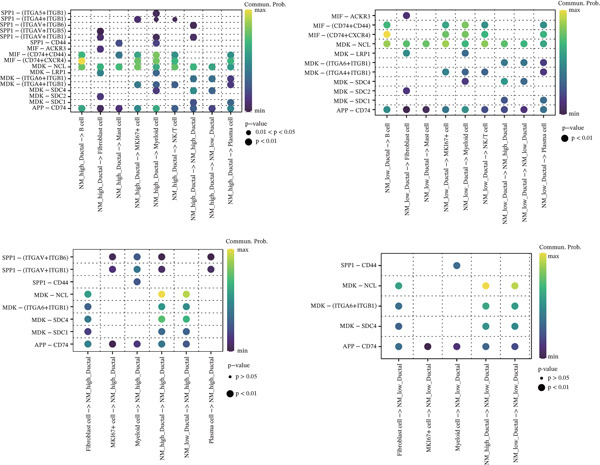
(D)

### 3.3. Transcriptomics Features of Nucleotide Metabolism–Related Genes in Pancreatic Cancer

Unsupervised clustering of pancreatic cancer patients from the TCGA cohort identified two distinct metabolic subtypes, C1 and C2 (Figure 6A). The C1 subtype exhibited higher nucleotide metabolism gene expression compared to C2. Furthermore, significant clinical differences were observed between the two subtypes (Figure 6B). The GSVA algorithm demonstrated notably elevated nucleotide metabolism activity in the C1 subtype relative to C2 (Figure 6C). Patients of the C1 subtype showed a significantly poorer prognosis compared to the C2 subtype, underscoring the adverse clinical impact of elevated nucleotide metabolism in pancreatic cancer (Figure 6D). Targeting inhibition of nucleotide metabolism may offer a promising therapeutic approach. Additionally, C2 subtype patients exhibited active bile acid metabolism, angiogenesis, and activation of Hedgehog signaling pathways, whereas C1 subtype patients displayed enhanced P53 signaling and glycolysis pathways (Figure 6E).

Figure 6Identification of clinical subtypes of pancreatic cancer based on nucleotide metabolism characteristics.  ^∗^ *p* < 0.05 and  ^∗∗^ *p* < 0.01. (A) Unsupervised hierarchical clustering. (B) Heatmap showing the expression characteristics of nucleotide metabolism genes in different clinical subtypes. (C) Comparison of nucleotide metabolism activity among different clinical subtypes. (D) Prognostic differences among different clinical subtypes. (E) Differences in HALLMARK signaling activity among different clinical subtypes.

**Figure:**
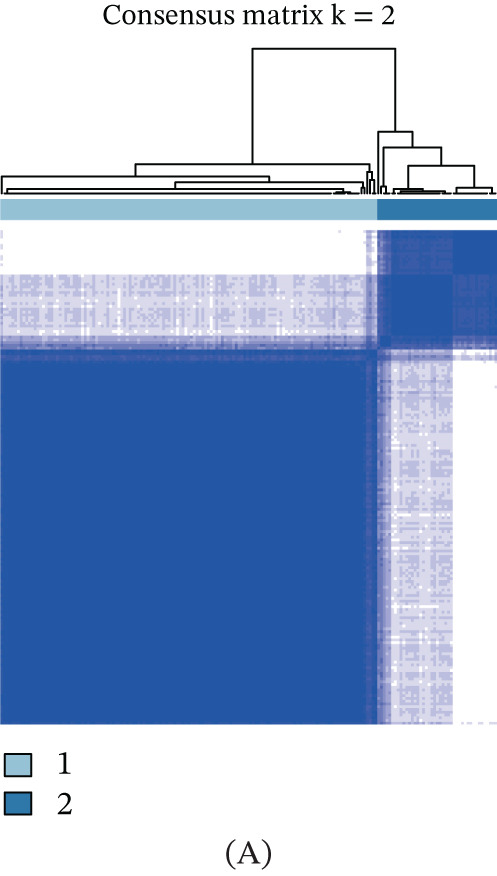
(A)

**Figure:**
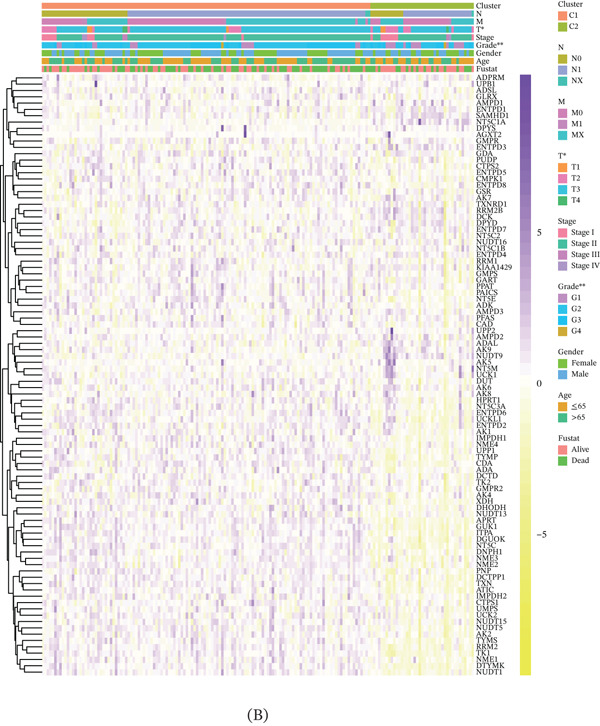
(B)

**Figure:**
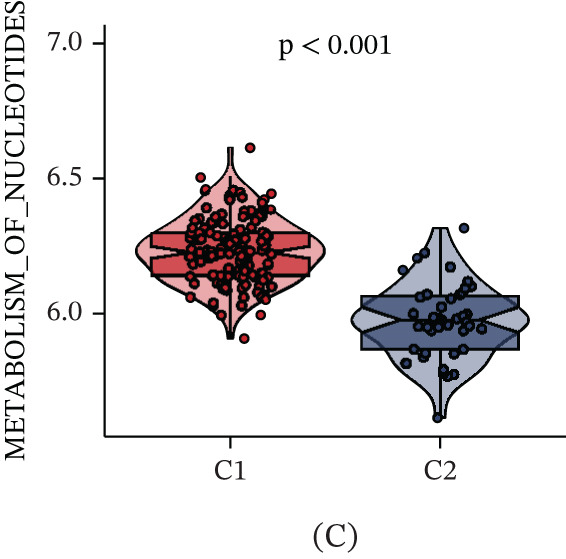
(C)

**Figure:**
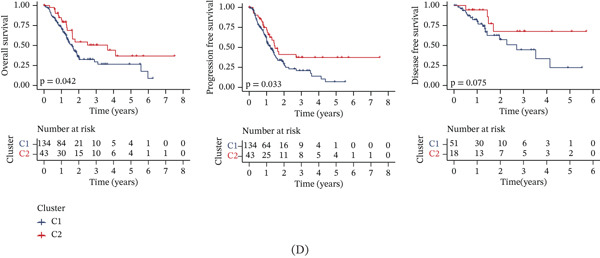
(D)

**Figure:**
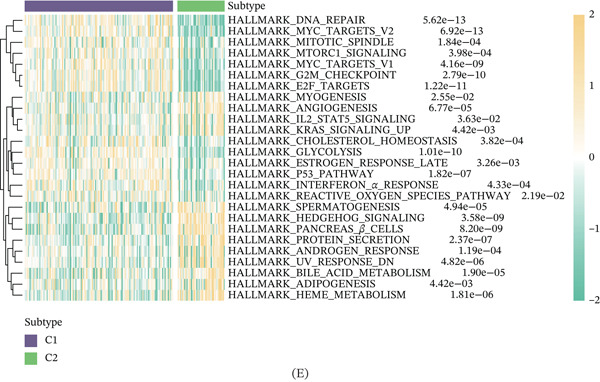
(E)

**Figure 7:**
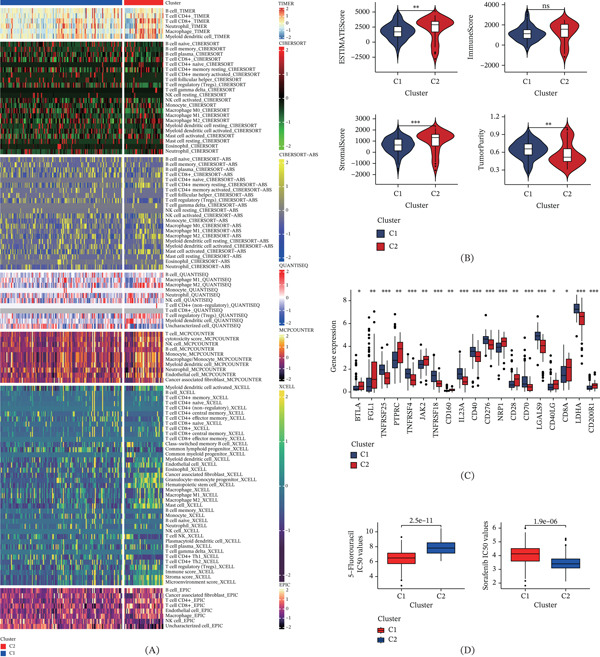
Differences in the immune microenvironment among clinical subtypes. ns, not significant;  ^∗^ *p* < 0.05,  ^∗∗^ *p* < 0.01, and  ^∗∗∗^ *p* < 0.001. (A) Assessment of cell infiltration in C1 and C2 subtypes using multiple immune cell infiltration prediction algorithms. (B) Evaluation of tumor purity and immune scores in C1 and C2 subtypes using the ESTIMATE algorithm. (C) Differential expression of immune checkpoints between C1 and C2 subtypes. (D) Potential beneficial drugs for C1 and C2 subtypes.

To further elucidate the relationship between nucleotide metabolism and immune microenvironment in pancreatic cancer, multiple immune algorithms, including ESTIMATE, TIMER, and CIBERSORT, were employed for comprehensive analysis. The results indicated that the C1 subtype, characterized by relatively active nucleotide metabolism, exhibited high tumor purity and low immune score (Figure 7B). Elevated tumor purity correlates with increased demand for pyrimidine and purine‐related metabolites, necessitating enhanced nucleotide metabolism to sustain this feature. Figure 7A provides a detailed examination of immune cell infiltration. Across all algorithms, consistent results were observed, suggesting that the C1 subtype exhibits reduced immune cell infiltration. Conversely, pancreatic cancer specimens from the C2 subtype showed a higher abundance of immune cell infiltration. Immune checkpoints, key regulators inhibiting immune‐mediated tumor elimination, were found to differ significantly in expression levels between the C1 and C2 groups. Higher expression of genes such as IL23A, CD40, CD276, and CD70 was observed in the C1 subtype, while elevated expression of JAK2, CD28, and CD8A was noted in the C2 subtype (Figure 7C). Drug susceptibility analysis revealed that 5‐fluorouracil was more beneficial for pancreatic cancer patients with active nucleotide metabolism, whereas sorafenib appeared more suitable for those with inactive nucleotide metabolism (Figure 7D).

**Figure 8:**
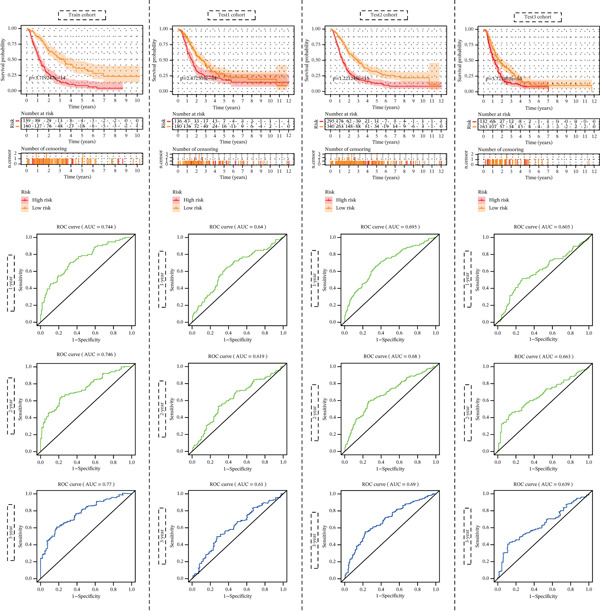
Survival curves and ROC curves of the prognostic panel.

LASSO‐Cox regression analysis determined a 10‐gene prognostic panel (Supporting Information 3: Figure S3A,B). These 10 genes included ENTPD5, DPYS, ADA, NT5E, IMPDH2, TXN, NME3, NT5M, CDA, and TK1 (Supporting Information 3: Figure S3B). Figure 8 exhibits good performance in predicting clinical outcomes of patients.

### 3.4. Identification of AK4 as a Central Nucleotide Metabolism Gene in Pancreatic Cancer

**Figure 9:**
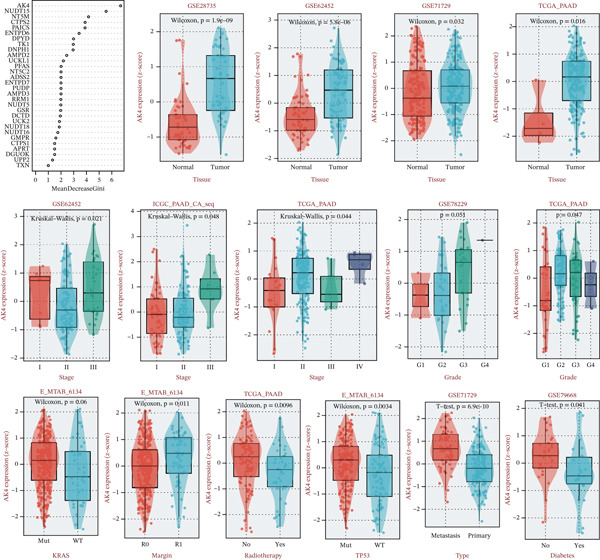
Clinical characteristic analysis of the key nucleotide metabolism pathway gene AK4.

**Figure 10:**
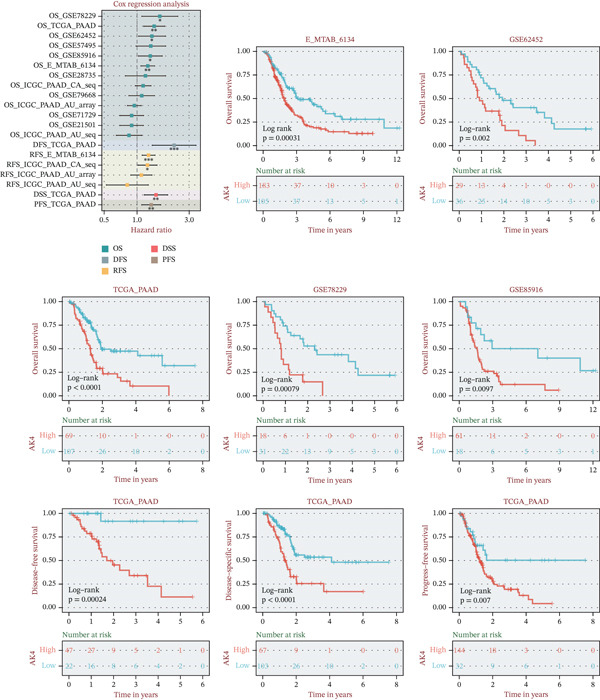
Prognostic analysis of the key nucleotide metabolism pathway gene AK4.  ^∗^ *p* < 0.05,  ^∗∗^ *p* < 0.01, and  ^∗∗∗^ *p* < 0.001.

The random forest algorithm identified AK4 as the most significant gene in nucleotide metabolism, ranking it first among all evaluated genes (Figure 9). In pancreatic cancer tissues, AK4 transcription levels were notably elevated compared to normal pancreatic tissue, with expression strongly correlating with clinical stage and grade (Figure 9). Elevated AK4 expression was also observed in patients with KRAS and TP53 mutations. Furthermore, the transcriptional profile of AK4 was associated with tumor metastasis and response to radiotherapy (Figure 9). Figure 9 illustrates the correlation between AK4 transcription levels and clinical outcomes in pancreatic cancer patients, with high AK4 expression linked to poorer prognosis (Figure 10).

### 3.5. Effect of AK4 on Pancreatic Cancer Cell Proliferation and Migration

**Figure 11:**
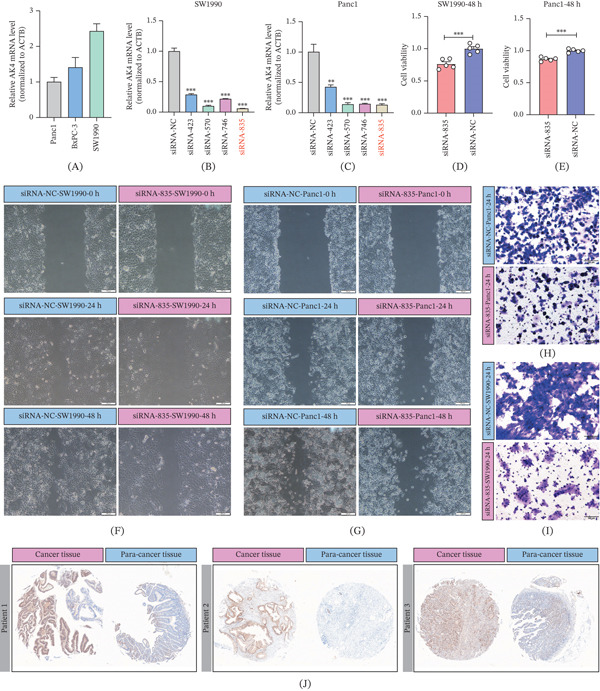
Effects of AK4 on the proliferation and migration of pancreatic cancer cell lines.  ^∗∗^ *p* < 0.01 and  ^∗∗∗^ *p* < 0.001. (A) Relative expression levels of AK4 in different pancreatic cancer cell lines. (B) Selection of the optimal siRNA for AK4 knockdown in SW1990 cells. (C) Selection of the optimal siRNA for AK4 knockdown in PANC1 cells. (D) CCK8 assay validating the effect of AK4 silencing on SW1990 cell proliferation. (E) CCK8 assay validating the effect of AK4 silencing on PANC1 cell proliferation. (F) Wound healing assay validating the effect of AK4 silencing on SW1990 cell migration. (G) Wound healing assay validating the effect of AK4 silencing on PANC1 cell migration. (H) Transwell assay validating the effect of AK4 silencing on SW1990 cell migration. (I) Transwell assay validating the effect of AK4 silencing on PANC1 cell migration. (J) Immunohistochemical analysis of AK4 expression characteristics.

Figure 11A reveals that AK4 expression was most pronounced in SW1990 cells. To assess the functional impact of AK4 on pancreatic cancer cells, an AK4 knockdown experiment was conducted in SW1990 and PANC1 cells. As depicted in Figure 11B,C, the siRNA‐835 sequence exhibited the most efficient knockdown of AK4 expression, making it the selected sequence for subsequent experiments. CCK8 assay results demonstrated that AK4 knockdown significantly suppressed SW1990 and PANC1 cell proliferation (Figure 11D,E). Additionally, wound healing assay results indicated that AK4 knockdown reduced the migratory capacity of SW1990 and PANC1 cells (Figure 11F,G). The Transwell assay also indicated the impact of the AK4 gene on the migratory capacity of SW1990 and PANC1 cells (Figure 11H,I). Immunohistochemical staining of tissue microarrays further confirmed that AK4 expression was markedly higher in pancreatic cancer tissue compared to adjacent normal tissue (Figure 11J).

## 4. Discussion

Pancreatic cancer remains one of the most lethal malignancies, with a 500‐year survival rate lingering around only 12% despite recent medical advances [40]. This dismal prognosis reflects the aggressive biology of pancreatic cancer, including its capacity for metabolic reprogramming to support unrestrained growth. It is well known that cancer cells modulate their microenvironment and metabolism to fulfill increased demands for energy and biosynthetic precursors [41]. Among these reprogrammed pathways, nucleotide metabolism has garnered increasing attention as a driver of tumor proliferation and survival [42]. Nucleotides are fundamental not only for DNA and RNA synthesis but also for energy transfer and signaling processes [43]. In pancreatic cancer, aberrant nucleotide metabolism is emerging as a pivotal factor that facilitates rapid tumor cell proliferation and contributes to therapeutic resistance [12, 44]. Beyond supporting cell‐intrinsic needs, deregulated nucleotide pathways can influence the TME; for example, accumulating evidence indicates that excessive nucleotide turnover in tumors can lead to immunosuppressive concentrations of metabolites (such as adenosine), thereby blunting antitumor immune responses.

Nucleotides, as essential macromolecules that carry biological information, primarily serve as precursors for nucleic acid synthesis and support cellular proliferation [45]. Ongoing studies on purines and pyrimidines have expanded the understanding of nucleotides, revealing their broader roles beyond promoting cell growth, including nonproliferative effects in tumor biology [46, 47]. Metabolomics analyses conducted in this study further substantiate the involvement of nucleotide metabolism in pancreatic cancer, as evidenced by the significantly elevated abundance of nucleotide‐related metabolites in cancerous tissue compared to normal pancreatic tissue.

Our single‐cell and spatial transcriptomics analyses shed light on how nucleotide metabolism varies across different cellular compartments of the tumor and between patients. We found that within the TME, MKI67^+^ proliferative tumor cells and infiltrating myeloid immune cells were among the subsets with the most active nucleotide metabolic gene signatures. In fact, the demand for nucleotides in these populations surpassed that of all other cell types analyzed, echoing observations by others that both cancer cells and certain immune cells exhibit high nucleotide turnover in the TME [41, 48, 49]. Tumor cells, especially those in a rapid growth phase, rely on increased nucleotide biosynthesis, whereas immune cells (such as activated T cells or macrophages) require nucleotides for clonal expansion and effector functions.

Compared to normal pancreatic tissue, cancerous tissues exhibited a marked increase in nucleotide metabolism across various cell types. Given that pancreatic cancer primarily originates in the pancreatic duct and is predominantly characterized by ductal adenocarcinoma, this study specifically focused on the nucleotide metabolism of ductal cells. However, the relatively small number of ductal cells in the single‐cell dataset limited the ability to thoroughly investigate their metabolic characteristics, which represents a limitation of the current study. Future research with larger single‐cell datasets will be necessary to more comprehensively explore the nucleotide metabolism profiles of ductal cells.

Importantly, our integrative analysis revealed significant interpatient heterogeneity in nucleotide metabolism, leading us to define two distinct metabolic subtypes of pancreatic cancer. Subtype C1 was characterized by hyperactive nucleotide metabolic pathways, whereas subtype C2 exhibited comparatively lower nucleotide metabolic activity. Clinically, these metabolic subtypes showed markedly different behaviors. Patients in the C1 group tended to present with more advanced disease and had a significantly worse prognosis than those in C2. In our cohort, C1 tumors were enriched for high tumor cell purity (i.e., a higher fraction of tumor cells relative to stromal/immune components) and demonstrated a paucity of infiltrating immune cells. In contrast, subtype C2 had relatively greater immune cell presence. Therefore, the C1 metabolic phenotype (a high‐nucleotide, “metabolically hot” tumor) may not only fuel aggressive growth but also underlie immune evasion. One plausible explanation is that the overactive nucleotide metabolism in C1 tumors leads to the accumulation of metabolites (like ATP, UTP, and their breakdown products) that can impair antitumor immunity or indicate a more hypoxic, adenosine‐rich milieu that discourages effective immune cell function. These characteristics likely contribute to the poorer outcomes observed in C1 patients. By contrast, the C2 subtype might resemble a less aggressive metabolic state, potentially allowing more robust immune surveillance and, hence, a better prognosis.

Prior studies in other cancers have identified metabolic gene expression signatures associated with differential prognosis and therapy responses [50, 51]. Our results suggest that assessing nucleotide metabolic activity could be an important addition to the molecular stratification of pancreatic cancer. In practical terms, patients with a C1‐like profile might benefit from more aggressive treatment or from therapies targeting metabolic pathways, whereas C2 patients might have a different risk profile. Future research should validate these subtypes in independent cohorts and explore whether they have predictive value for treatment.

A central finding of our multiomics study is the identification of AK4 as a key regulatory gene in the nucleotide metabolism network of pancreatic cancer. AKs are a family of enzymes that maintain cellular energy homeostasis by catalyzing phosphate transfer between adenine nucleotides [52]. As a member of the AK family, AK4 is essential for cellular energy metabolism and the maintenance of adenine nucleotide homeostasis across various subcellular compartments [53]. AK4 has been shown to interact with ADP/ATP translocase, indirectly modulating mitochondrial membrane permeability, suggesting its involvement in mitochondrial function regulation [54].

In recent years, the role of AK4 in malignant tumors has garnered increasing attention. Xin et al. [17] utilized immunohistochemistry and molecular biology techniques to demonstrate that elevated AK4 expression is closely associated with tumor stage, distant metastasis, and vascular invasion in bladder cancer, as well as with a poor prognosis in affected patients. In vitro experiments further established that AK4 enhanced the proliferation, invasion, and metastasis of bladder cancer cells. Zhang et al. [55] also reported that AK4 promoted the progression of HER2‐positive breast cancer. Additionally, Jan et al. [19] observed a significant increase in AK4 expression in lung adenocarcinoma tissue compared to normal lung tissue, with this overexpression correlating with tumor stage and poor prognosis. Furthermore, Jan et al. [19] demonstrated that AK4 knockdown inhibited the invasive potential of lung cancer cell lines, whereas AK4 overexpression promoted invasion both in vitro and in vivo. This study also showed that knockdown of AK4 expression suppressed the proliferation and migration of pancreatic cancer cell lines. In conclusion, AK4 is overexpressed in various malignant tumors and promotes tumor cell invasion and metastasis through multiple mechanisms, supporting its potential as a therapeutic target.

## 5. Conclusion

Nucleotide metabolism plays a significant role in the initiation and progression of pancreatic cancer. MKI67^+^ and myeloid cells represent the primary cell types with active nucleotide metabolism. Knockdown of AK4, a key gene in nucleotide metabolism, leads to a reduction in the proliferation and migration of pancreatic cancer cells to some degree.

## Author Contributions

All authors are solely responsible for the content and writing of the manuscript. The research design was determined through joint discussions among all authors. Jun Li and Yiqun Yao contributed to this study equally.

## Funding

No funding was received for this manuscript.

## Ethics Statement

The authors have nothing to report.

## Consent

The authors have nothing to report.

## Conflicts of Interest

The authors declare no conflicts of interest.

## Supporting Information

Additional supporting information can be found online in the Supporting Information section.

## Supporting information


**Supporting Information 1** Figure S1: Single‐cell data analysis identifying 25 potential cell subpopulations in pancreatic cancer.

**Supporting Information 2** Figure S2: UMAP plots of nucleotide metabolism characteristics in various cells, including Add, AUCell, singscore, ssgsea, UCell, and Scoring algorithms.

**Supporting Information 3** Figure S3: LASSO‐Cox regression analysis. (A) LASSO analysis. (B) Multivariable Cox regression analysis.  ^∗^ *p* < 0.05,  ^∗∗^ *p* < 0.01, and  ^∗∗∗^ *p* < 0.001.

**Supporting Information 4** Table S1: Primer sequences. Table S2: Gene silencing sequences.

## Data Availability

The datasets analyzed in this work may be found in the Supporting Information section or by contacting the corresponding authors.

## References

[1] Xue K. , Huang X. , Zhao P. , Zhang Y. , and Tian B. , Perioperative and Long-Term Survival Outcomes of Pancreatectomy With Arterial Resection in Borderline Resectable or Locally Advanced Pancreatic Cancer Following Neoadjuvant Therapy: A Systematic Review and Meta-Analysis, International Journal Of Surgery. (2023) 109, no. 12, 4309–4321, 10.1097/JS9.0000000000000742, 38259002. 38259002 PMC10720779

[2] Zhang Y. , Zhang H. , Yang Y. , Wu C. , Zhang L. , Xia W. , Wang X. , Zhang X. , Cao L. , Liu M. , Zhang J. , Yan F. , Shen B. , and Wen N. , A Clinically Validated 3D Deep Learning Approach for Quantifying Vascular Invasion in Pancreatic Cancer, NPJ Digital Medicine. (2026) 9, no. 1, 10.1038/s41746-025-02260-3, 41476122. PMC12848109 41476122

[3] Mannucci A. and Goel A. , Advances in Pancreatic Cancer Early Diagnosis, Prevention, and Treatment: The Past, the Present, and the Future, A Cancer Journal for Clinicians. (2026) 76, no. 1, 10.3322/caac.70035, e70035, 40971231. PMC12788368 40971231

[4] Park J. , Patterson J. , Acitores Cortina J. M. , Gu T. , Hur C. , and Tatonetti N. , Enhancing EHR-Based Pancreatic Cancer Prediction With LLM-Derived Embeddings, NPJ Digital Medicine. (2025) 8, no. 1, 10.1038/s41746-025-01869-8, 40691317. PMC12280092 40691317

[5] Yuan Q. , Sun J. , Hong Z. , and Shang D. , Determining a Robust Prognostic Biomarker for 804 Patients With Pancreatic Cancer Using a Machine Learning Computational Framework, International Journal Of Surgery. (2025) 111, no. 1, 1561–1563, 10.1097/JS9.0000000000002016, 39166940. 39166940 PMC11745771

[6] Li B. , Wang B. , Zhuang P. , Cao H. , Wu S. , Tan Z. , Gao S. , Li P. , Jing W. , Shao Z. , Zheng K. , Wu L. , Gao B. , Wang Y. , Jiang H. , Guo S. , He L. , Yang Y. , and Jin G. , A Novel Staging System Derived From Natural Language Processing of Pathology Reports to Predict Prognostic Outcomes of Pancreatic Cancer: A Retrospective Cohort Study, International Journal Of Surgery. (2023) 109, no. 11, 3476–3489, 10.1097/JS9.0000000000000648, 37578452. 37578452 PMC10651292

[7] Caputo D. , Quagliarini E. , Coppola A. , La Vaccara V. , Marmiroli B. , Sartori B. , Caracciolo G. , and Pozzi D. , Inflammatory Biomarkers and Nanotechnology: New Insights in Pancreatic Cancer Early Detection, International Journal Of Surgery. (2023) 109, no. 10, 2934–2940, 10.1097/JS9.0000000000000558, 37352522. 37352522 PMC10583897

[8] Xu C. , Jun E. , Okugawa Y. , Toiyama Y. , Borazanci E. , Bolton J. , Taketomi A. , Kim S. C. , Shang D. , von Hoff D. , Zhang G. , and Goel A. , A Circulating Panel of circRNA Biomarkers for the Noninvasive and Early Detection of Pancreatic Ductal Adenocarcinoma, Gastroenterology. (2024) 166, no. 1, 178–190.e16, 10.1053/j.gastro.2023.09.050, 37839499. 37839499 PMC10843014

[9] Su Y. Y. , Chao Y. J. , Wang C. J. , Liao T. K. , Su P. J. , Huang C. J. , Chiang N. J. , Yu Y. T. , Tsai H. M. , Chen L. T. , and Shan Y. S. , The Experience of Neoadjuvant Chemotherapy Versus Upfront Surgery in Resectable Pancreatic Cancer: A Cross Sectional Study, International Journal Of Surgery. (2023) 109, no. 9, 2614–2623, 10.1097/JS9.0000000000000495, 37300888. 37300888 PMC10498854

[10] Fu N. , Fu W. , Chen H. , Chai W. , Qian X. , Wang W. , Jiang Y. , and Shen B. , A Deep-Learning Radiomics-Based Lymph Node Metastasis Predictive Model for Pancreatic Cancer: A Diagnostic Study, International Journal Of Surgery. (2023) 109, no. 8, 2196–2203, 10.1097/JS9.0000000000000469, 37216230. 37216230 PMC10442094

[11] Tang Y. , Su Y. X. , Zheng J. M. , Zhuo M. L. , Qian Q. F. , Shen Q. L. , Lin P. , and Chen Z. K. , Radiogenomic Analysis for Predicting Lymph Node Metastasis and Molecular Annotation of Radiomic Features in Pancreatic Cancer, Journal Of Translational Medicine. (2024) 22, no. 1, 10.1186/s12967-024-05479-y, 39075486. PMC11288107 39075486

[12] Liu Q. , Liu J. , Wang S. , Bao N. , Zhao X. , and Wang L. , Roles of Nucleotide Metabolism in Pancreatic Cancer, Frontiers In Immunology. (2025) 16, 1637768, 10.3389/fimmu.2025.1637768. 41200180 PMC12585966

[13] Dzeja P. and Terzic A. , Adenylate Kinase and AMP Signaling Networks: Metabolic Monitoring, Signal Communication and Body Energy Sensing, International Journal Of Molecular Sciences. (2009) 10, no. 4, 1729–1772, 10.3390/ijms10041729, 2-s2.0-67149111580, 19468337. 19468337 PMC2680645

[14] Liu H. , Pu Y. , Amina Q. , Wang Q. , Zhang M. , Song J. , Guo J. , and Mardan M. , Prognostic and Therapeutic Potential of Adenylate Kinase 2 in Lung Adenocarcinoma, Scientific Reports. (2019) 9, no. 1, 17757, 10.1038/s41598-019-53594-4, 31780678. 31780678 PMC6883075

[15] Panayiotou C. , Solaroli N. , Johansson M. , and Karlsson A. , Evidence of an Intact N-Terminal Translocation Sequence of Human Mitochondrial Adenylate Kinase 4, International Journal of Biochemistry & Cell Biology. (2010) 42, no. 1, 62–69, 10.1016/j.biocel.2009.09.007, 2-s2.0-70549098811, 19766732. 19766732

[16] Lanning N. J. , Looyenga B. D. , Kauffman A. L. , Niemi N. M. , Sudderth J. , DeBerardinis R. J. , and MacKeigan J. P. , A Mitochondrial RNAi Screen Defines Cellular Bioenergetic Determinants and Identifies an Adenylate Kinase as a Key Regulator of ATP Levels, Cell Reports. (2014) 7, no. 3, 907–917, 10.1016/j.celrep.2014.03.065, 2-s2.0-84899828061, 24767988. 24767988 PMC4046887

[17] Xin F. , Yao D. W. , Fan L. , Liu J. H. , and Liu X. D. , Adenylate Kinase 4 Promotes Bladder Cancer Cell Proliferation and Invasion, Clinical and Experimental Medicine.(2019) 19, no. 4, 525–534. 31463832 10.1007/s10238-019-00576-5

[18] Jan Y. H. , Lai T. C. , Yang C. J. , Lin Y. F. , Huang M. S. , and Hsiao M. , Adenylate Kinase 4 Modulates Oxidative Stress and Stabilizes HIF-1*α* to Drive Lung Adenocarcinoma Metastasis, Journal Of Hematology & Oncology. (2019) 12, no. 1, 10.1186/s13045-019-0698-5, 2-s2.0-85060673588, 30696468. PMC6352453 30696468

[19] Jan Y. H. , Tsai H. Y. , Yang C. J. , Huang M. S. , Yang Y. F. , Lai T. C. , Lee C. H. , Jeng Y. M. , Huang C. Y. , Su J. L. , Chuang Y. J. , and Hsiao M. , Adenylate Kinase-4 Is a Marker of Poor Clinical Outcomes That Promotes Metastasis of Lung Cancer by Downregulating the Transcription Factor ATF3, Cancer Research. (2012) 72, no. 19, 5119–5129, 10.1158/0008-5472.CAN-12-1842, 2-s2.0-84867121913, 23002211. 23002211

[20] Huang M. , Qin X. , Wang Y. , and Mao F. , Identification of AK4 as a Novel Therapeutic Target for Serous Ovarian Cancer, Oncology Letters. (2020) 20, no. 6, 10.3892/ol.2020.12209, 33123257. PMC7583734 33123257

[21] Liu C. , Qin H. , Liu H. , Wei T. , Wu Z. , Shang M. , Liu H. , Wang A. , Liu J. , Shang D. , and Yin P. , Tissue Metabolomics Identified New Biomarkers for the Diagnosis and Prognosis Prediction of Pancreatic Cancer, Frontiers In Oncology. (2022) 12, 991051, 10.3389/fonc.2022.991051, 36119530. 36119530 PMC9479084

[22] Yousuf S. , Qiu M. , Voith von Voithenberg L. , Hulkkonen J. , Macinkovic I. , Schulz A. R. , Hartmann D. , Mueller F. , Mijatovic M. , Ibberson D. , AlHalabi K. T. , Hetzer J. , Anders S. , Brüne B. , Mei H. E. , Imbusch C. D. , Brors B. , Heikenwälder M. , Gaida M. M. , Büchler M. W. , Weigert A. , Hackert T. , and Roth S. , Spatially Resolved Multi-Omics Single-Cell Analyses Inform Mechanisms of Immune Dysfunction in Pancreatic Cancer, Gastroenterology. (2023) 165, no. 4, 891–908.e14, 10.1053/j.gastro.2023.05.036, 37263303. 37263303

[23] Wang Q. , Ni Y. , Lu S. , Zhang B. , Ji J. , Cai Q. , Yan C. , Qi F. , Shi M. , and Zhang J. , Multi-Dimensional Omics Integrated Machine Learning Framework Identifies Macrophage-Fibroblast-Tumor Co-Infiltration Patterns to Predict Prognosis in Gastric Cancer, NPJ Digital Medicine. (2026) 9, no. 1, 10.1038/s41746-025-02179-9, 41360923. PMC12775079 41360923

[24] Peng J. , Sun J. , Yu Y. , Yuan Q. , and Zhang Y. , Integrative Multi-Omics Analysis Reveals the Role of Toll-Like Receptor Signaling in Pancreatic Cancer, Scientific Reports. (2025) 15, no. 1, 10.1038/s41598-024-84062-3, 39747201. PMC11696379 39747201

[25] Li X. , Guan H. , Ma C. , Dai Y. , Su J. , Chen X. , Yuan Q. , and Wang J. , Combination of Bulk RNA Sequencing and scRNA Sequencing Uncover the Molecular Characteristics of MAPK Signaling in Kidney Renal Clear Cell Carcinoma, Aging. (2024) 16, no. 2, 1414–1439, 10.18632/aging.205436, 38217548. 38217548 PMC10866414

[26] He S. , Sun J. , Guan H. , Su J. , Chen X. , Hong Z. , and Wang J. , Molecular Characteristics and Prognostic Significances of Lysosomal-Dependent Cell Death in Kidney Renal Clear Cell Carcinoma, Aging. (2024) 16, no. 5, 4862–4888, 10.18632/aging.205639, 38460947. 38460947 PMC10968703

[27] Trapnell C. , Cacchiarelli D. , Grimsby J. , Pokharel P. , Li S. , Morse M. , Lennon N. J. , Livak K. J. , Mikkelsen T. S. , and Rinn J. L. , The Dynamics and Regulators of Cell Fate Decisions Are Revealed by Pseudotemporal Ordering of Single Cells, Nature Biotechnology. (2014) 32, no. 4, 381–386, 10.1038/nbt.2859, 2-s2.0-84900873950, 24658644. PMC4122333 24658644

[28] Jin S. , Guerrero-Juarez C. F. , Zhang L. , Chang I. , Ramos R. , Kuan C. H. , Myung P. , Plikus M. V. , and Nie Q. , Inference and Analysis of Cell-Cell Communication Using CellChat, Nature Communications. (2021) 12, no. 1, 10.1038/s41467-021-21246-9, 33597522. PMC7889871 33597522

[29] Chen D. T. , Davis-Yadley A. H. , Huang P. Y. , Husain K. , Centeno B. A. , Permuth-Wey J. , Pimiento J. M. , and Malafa M. , Prognostic Fifteen-Gene Signature for Early Stage Pancreatic Ductal Adenocarcinoma, PLOS One. (2015) 10, no. 8, e0133562, 10.1371/journal.pone.0133562, 2-s2.0-84941949680, 26247463. 26247463 PMC4527782

[30] Zhang G. , He P. , Tan H. , Budhu A. , Gaedcke J. , Ghadimi B. M. , Ried T. , Yfantis H. G. , Lee D. H. , Maitra A. , Hanna N. , Alexander H. R. , and Hussain S. P. , Integration of Metabolomics and Transcriptomics Revealed a Fatty Acid Network Exerting Growth Inhibitory Effects in Human Pancreatic Cancer, Clinical Cancer Research: An Official Journal of the American Association for Cancer Research. (2013) 19, no. 18, 4983–4993, 10.1158/1078-0432.CCR-13-0209, 2-s2.0-84884570719, 23918603. 23918603 PMC3778077

[31] Yang S. , He P. , Wang J. , Schetter A. , Tang W. , Funamizu N. , Yanaga K. , Uwagawa T. , Satoskar A. R. , Gaedcke J. , Bernhardt M. , Ghadimi B. M. , Gaida M. M. , Bergmann F. , Werner J. , Ried T. , Hanna N. , Alexander H. R. , and Hussain S. P. , A Novel MIF Signaling Pathway Drives the Malignant Character of Pancreatic Cancer by Targeting NR3C2, Cancer Research. (2016) 76, no. 13, 3838–3850, 10.1158/0008-5472.CAN-15-2841, 2-s2.0-84977570870, 27197190. 27197190 PMC4930741

[32] Puleo F. , Nicolle R. , Blum Y. , Cros J. , Marisa L. , Demetter P. , Quertinmont E. , Svrcek M. , Elarouci N. , Iovanna J. , Franchimont D. , Verset L. , Galdon M. G. , Devière J. , de Reyniès A. , Laurent-Puig P. , van Laethem J. L. , Bachet J. B. , and Maréchal R. , Stratification of Pancreatic Ductal Adenocarcinomas Based on Tumor and Microenvironment Features, Gastroenterology. (2018) 155, no. 6, 1999–2013.e3, 10.1053/j.gastro.2018.08.033, 2-s2.0-85056697172, 30165049. 30165049

[33] Liberzon A. , Birger C. , Thorvaldsdóttir H. , Ghandi M. , Mesirov J. P. , and Tamayo P. , The Molecular Signatures Database (MSigDB) Hallmark Gene Set Collection, Cell Systems. (2015) 1, no. 6, 417–425, 10.1016/j.cels.2015.12.004, 2-s2.0-84955328286, 26771021. 26771021 PMC4707969

[34] Xu C. , Sui S. , Shang Y. , Yu Z. , Han J. , Zhang G. , Ntim M. , Hu M. , Gong P. , Chen H. , and Zhang X. , The Landscape of Immune Cell Infiltration and Its Clinical Implications of Pancreatic Ductal Adenocarcinoma, Journal of Advanced Research. (2020) 24, 139–148, 10.1016/j.jare.2020.03.009, 32322419. 32322419 PMC7171261

[35] Yoshihara K. , Shahmoradgoli M. , Martínez E. , Vegesna R. , Kim H. , Torres-Garcia W. , Treviño V. , Shen H. , Laird P. W. , Levine D. A. , Carter S. L. , Getz G. , Stemke-Hale K. , Mills G. B. , and Verhaak R. G. W. , Inferring Tumour Purity and Stromal and Immune Cell Admixture From Expression Data, Nature Communications. (2013) 4, no. 1, 10.1038/ncomms3612, 2-s2.0-84885673911, 24113773. PMC3826632 24113773

[36] Li T. , Fu J. , Zeng Z. , Cohen D. , Li J. , Chen Q. , Li B. , and Liu X. S. , TIMER2.0 for Analysis of Tumor-Infiltrating Immune Cells, Nucleic Acids Research. (2020) 48, no. W1, W509–W514, 10.1093/nar/gkaa407, 32442275. 32442275 PMC7319575

[37] Li T. , Fan J. , Wang B. , Traugh N. , Chen Q. , Liu J. S. , Li B. , and Liu X. S. , TIMER: A Web Server for Comprehensive Analysis of Tumor-Infiltrating Immune Cells, Cancer Research. (2017) 77, no. 21, e108–e110, 10.1158/0008-5472.CAN-17-0307, 2-s2.0-85035064069, 29092952. 29092952 PMC6042652

[38] Maeser D. , Gruener R. F. , and Huang R. S. , oncoPredict: An R Package for Predicting In Vivo or Cancer Patient Drug Response and Biomarkers From Cell Line Screening Data, Briefings In Bioinformatics. (2021) 22, no. 6, 10.1093/bib/bbab260, 34260682. PMC8574972 34260682

[39] Liu Z. , Liu L. , Weng S. , Xu H. , Xing Z. , Ren Y. , Ge X. , Wang L. , Guo C. , Li L. , Cheng Q. , Luo P. , Zhang J. , and Han X. , BEST: A Web Application for Comprehensive Biomarker Exploration on Large-Scale Data in Solid Tumors, Journal Of Big Data. (2023) 10, no. 1, 10.1186/s40537-023-00844-y.

[40] Liu J. , Yuan Q. , Chen X. , Yang Y. , Xie T. , Zhang Y. , Qi B. , Li S. , and Shang D. , Prognostic and Therapeutic Value of the Eph/Ephrin Signaling Pathway in Pancreatic Cancer Explored Based on Bioinformatics, Scientific Reports. (2024) 14, no. 1, 17650, 10.1038/s41598-024-68385-9. 39085301 PMC11291735

[41] Wu J. , Rong Y. , Li T. , Wilson C. M. , He Y. , Chen D. , Han J. , and Zhang X. , Editorial: Targeting Nucleotide Metabolism for Enhancing Antitumor Immunity, Frontiers In Immunology. (2024) 15, 1412057, 10.3389/fimmu.2024.1412057, 38715612. 38715612 PMC11074342

[42] Ma J. , Zhong M. , Xiong Y. , Gao Z. , Wu Z. , Liu Y. , and Hong X. , Emerging Roles of Nucleotide Metabolism in Cancer Development: Progress and Prospect, Aging. (2021) 13, no. 9, 13349–13358, 10.18632/aging.202962, 33952722. 33952722 PMC8148454

[43] Chandel N. S. , Nucleotide Metabolism, Cold Spring Harbor Perspectives In Biology. (2021) 13, no. 7, a040592, 10.1101/cshperspect.a040592, 34210662. 34210662 PMC8247561

[44] Ni C. , Liu W. , Zheng K. , Guo S. , Song B. , Jing W. , Li G. , Li B. , Ni C. , Shi K. , Jin G. , and Yu G. , PI3K/ c-Myc/AFF4 Axis Promotes Pancreatic Tumorigenesis Through Fueling Nucleotide Metabolism, International Journal Of Biological Sciences. (2023) 19, no. 6, 1968–1982, 10.7150/ijbs.77150, 37063434. 37063434 PMC10092763

[45] Rathbone M. P. , Middlemiss P. J. , Gysbers J. W. , DeForge S. , Costello P. , and Del Maestro R. F. , Purine Nucleosides and Nucleotides Stimulate Proliferation of a Wide Range of Cell Types, In Vitro Cellular & Developmental Biology : Journal of the Tissue Culture Association. (1992) 28, no. 7-8, 529–536, 10.1007/BF02634137, 2-s2.0-0026670251, 1522046. 1522046

[46] Siddiqui A. and Ceppi P. , A Non-Proliferative Role of Pyrimidine Metabolism in Cancer, Molecular Metabolism. (2020) 35, 100962, 10.1016/j.molmet.2020.02.005, 32244187. 32244187 PMC7096759

[47] Garavito M. F. , Narváez-Ortiz H. Y. , and Zimmermann B. H. , Pyrimidine Metabolism: Dynamic and Versatile Pathways in Pathogens and Cellular Development, Journal of Genetics and Genomics. (2015) 42, no. 5, 195–205, 10.1016/j.jgg.2015.04.004, 2-s2.0-84947042536, 26059768. 26059768

[48] Madsen H. B. , Peeters M. J. , Straten P. T. , and Desler C. , Nucleotide Metabolism in the Regulation of Tumor Microenvironment and Immune Cell Function, Current Opinion in Biotechnology. (2023) 84, 103008, 10.1016/j.copbio.2023.103008. 37863018

[49] Pavlova N. N. and Thompson C. B. , The Emerging Hallmarks of Cancer Metabolism, Cell Metabolism. (2016) 23, no. 1, 27–47, 10.1016/j.cmet.2015.12.006, 2-s2.0-84955326448, 26771115. 26771115 PMC4715268

[50] Yuan Q. , Deng D. , Pan C. , Ren J. , Wei T. , Wu Z. , Zhang B. , Li S. , Yin P. , and Shang D. , Integration of Transcriptomics, Proteomics, and Metabolomics Data to Reveal HER2-Associated Metabolic Heterogeneity in Gastric Cancer With Response to Immunotherapy and Neoadjuvant Chemotherapy, Frontiers in Immunology. (2022) 13, 951137, 10.3389/fimmu.2022.951137, 35990657. 35990657 PMC9389544

[51] Wei T. , Liu J. , Ma S. , Wang M. , Yuan Q. , Huang A. , Wu Z. , Shang D. , and Yin P. , A Nucleotide Metabolism-Related Gene Signature for Risk Stratification and Prognosis Prediction in Hepatocellular Carcinoma Based on an Integrated Transcriptomics and Metabolomics Approach, Metabolites. (2023) 13, no. 11, 10.3390/metabo13111116, 37999212. PMC10673507 37999212

[52] Wujak M. , Veith C. , Wu C. Y. , Wilke T. , Kanbagli Z. I. , Novoyatleva T. , Guenther A. , Seeger W. , Grimminger F. , Sommer N. , Schermuly R. T. , and Weissmann N. , Adenylate Kinase 4-A Key Regulator of Proliferation and Metabolic Shift in Human Pulmonary Arterial Smooth Muscle Cells via Akt and HIF-1*α* Signaling Pathways, International Journal of Molecular Sciences. (2021) 22, no. 19, 10371, 10.3390/ijms221910371, 34638712. 34638712 PMC8508902

[53] Panayiotou C. , Solaroli N. , and Karlsson A. , The Many Isoforms of Human Adenylate Kinases, International Journal of Biochemistry & Cell Biology. (2014) 49, 75–83, 10.1016/j.biocel.2014.01.014, 2-s2.0-84894239646, 24495878. 24495878

[54] Lu W. , Yang Z. , Wang M. , Li S. , Bi H. , and Yang X. , Identification and Verification of AK4 as a Protective Immune-Related Biomarker in Adipose-Derived Stem Cells and Breast Cancer, Heliyon. (2024) 10, no. 7, e27357, 10.1016/j.heliyon.2024.e27357, 38560200. 38560200 PMC10980947

[55] Zhang J. , Yin Y. T. , Wu C. H. , Qiu R. L. , Jiang W. J. , Deng X. G. , and Li Z. X. , AK4 Promotes the Progression of HER2-Positive Breast Cancer by Facilitating Cell Proliferation and Invasion, Disease Markers. (2019) 2019, 8186091, 10.1155/2019/8186091. 31827645 PMC6886328

