# Supplementary figures and images for: Multiomics Analysis of Nucleotide Metabolism Highlights the Important Role of Adenylate Kinase 4 in Pancreatic Cancer
Source: Hum Mutat. 2026 May 3;2026:7729933. doi: 10.1155/humu/7729933 (PMC13136522; doi:10.1155/humu/7729933)

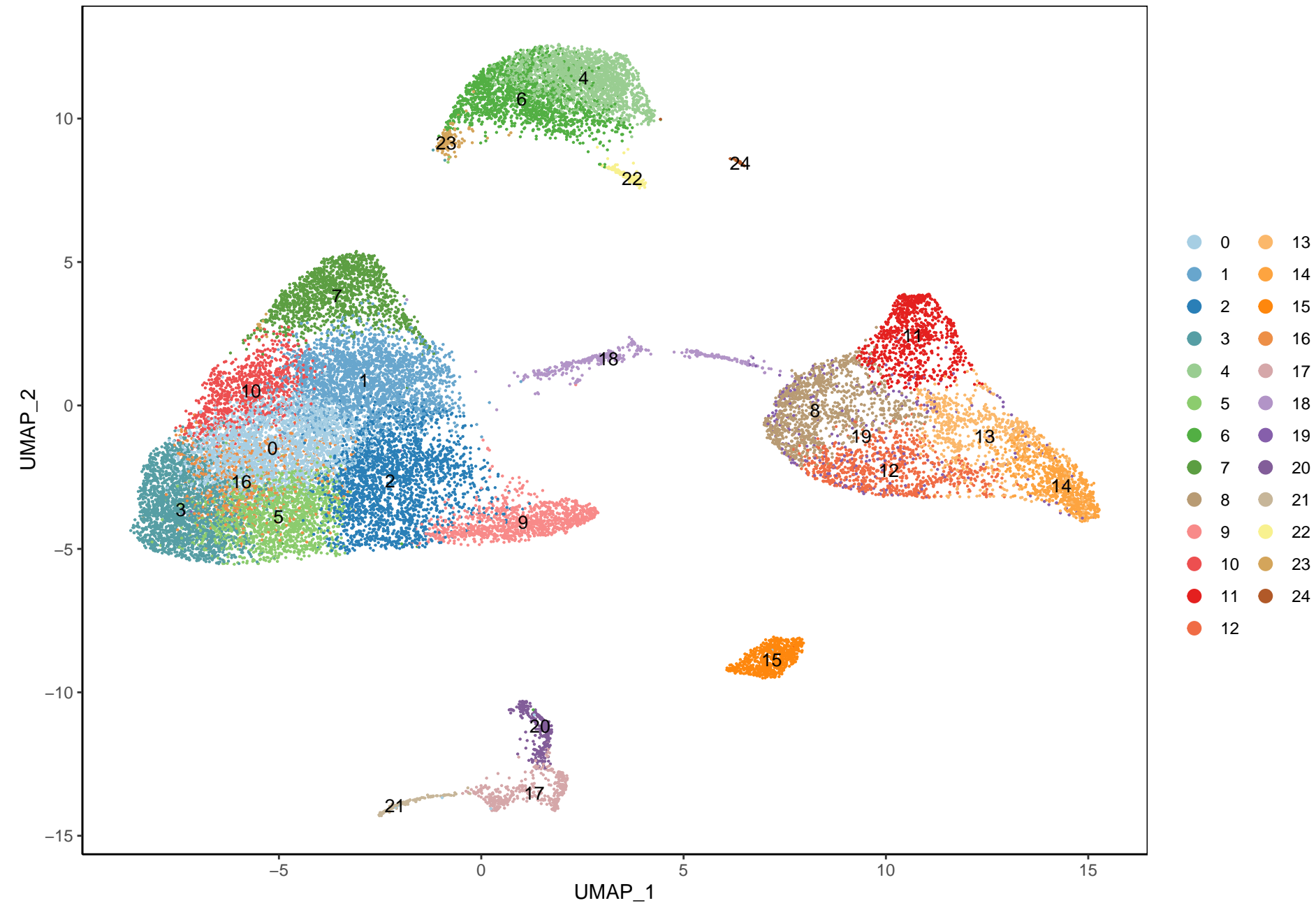

Supplement: Supplementary file 1 — Supporting Information 1 Figure S1: Single‐cell data analysis identifying 25 potential cell subpopulations in pancreatic cancer. [file HUMU-2026-7729933-s001.pdf]

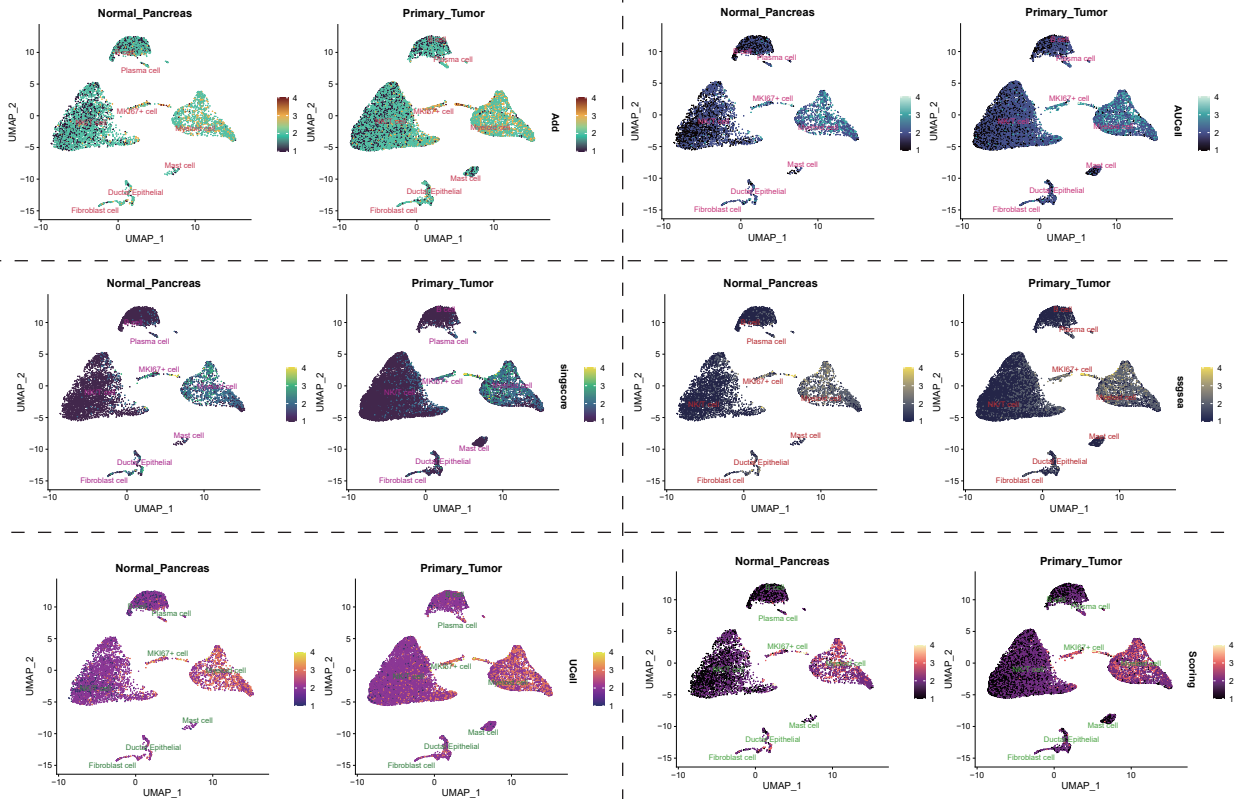

Supplement: Supplementary file 2 — Supporting Information 2 Figure S2: UMAP plots of nucleotide metabolism characteristics in various cells, including Add, AUCell, singscore, ssgsea, UCell, and Scoring algorithms. [file HUMU-2026-7729933-s003.pdf]

A

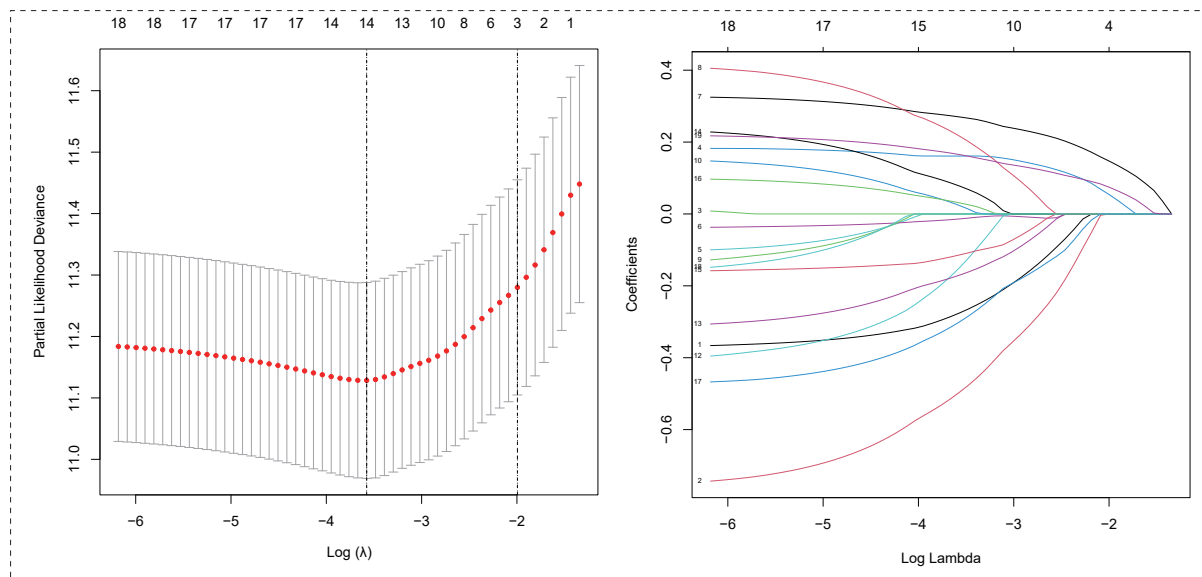

B

## Hazard ratio

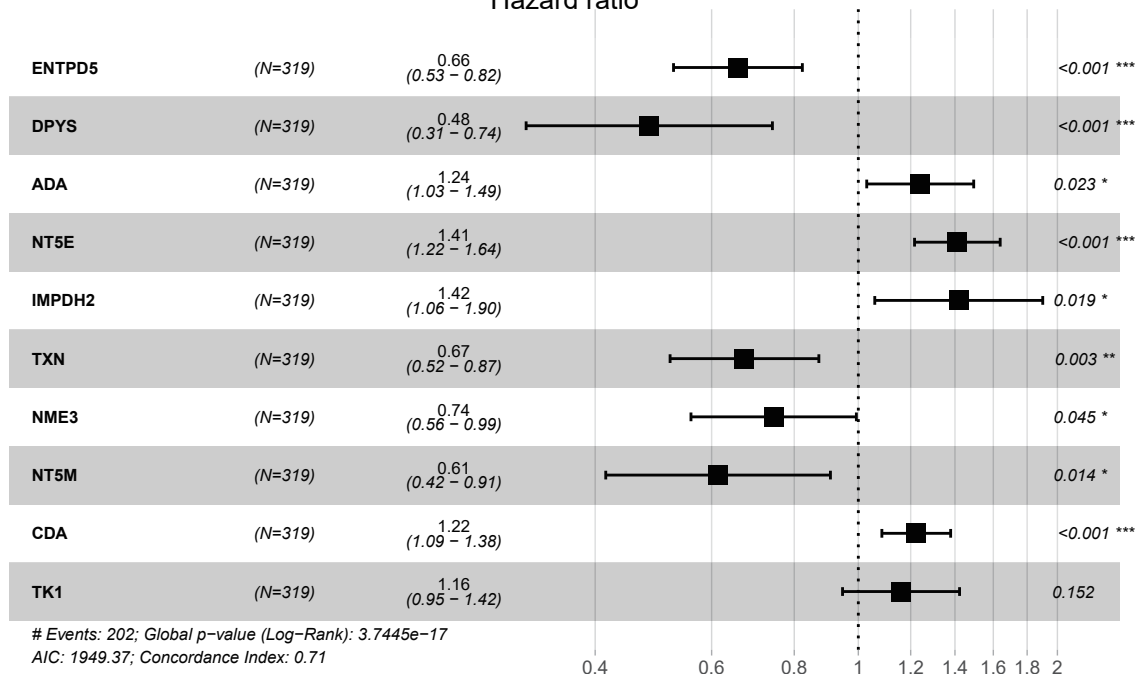

Supplement: Supplementary file 3 — Supporting Information 3 Figure S3: LASSO‐Cox regression analysis. (A) LASSO analysis. (B) Multivariable Cox regression analysis. ∗ p < 0.05, ∗∗ p < 0.01, and ∗∗∗ p < 0.001. [file HUMU-2026-7729933-s002.pdf]
